# Case of Rupture of the Uterus with Operation

**Published:** 1859-12

**Authors:** R. A. T. Ridley

**Affiliations:** LaGrange, Ga.


ATLANTA

Medical and Surgical Journal.

VOL. V.] DECEMBER, 1859. [No. 4.

### ORIGINAL COMMUNICATIONS.

### ARTICLE I.

*Case of Rupture of the Uterus with Operation.* By R A. T. RIDLEY, A. M. M. D., of LaGrange, Ga.

MESSRS. EDITORS—During the month of May last I treated a case of rare occurrence in the annals of Surgery, and believing that it would be interesting to the numerous readers of your widely circulated Journal, I concluded to report it for publication.

Lucinda, a negro woman, the property of E. A. REID, Esq., of Troup county, about twenty-four years old, healthy and strong, though of contracted Pelvis, was taken in Labor with her third child. Her two first Labors were difficult; I attended her in both. When she was first taken, in her third confinement, I was again called, but being absent, Dr. James A. Long, of LaGrange attended. At first nothing uncommon occurred save the discharge of the Liquor Amnii, which commenced when the Labor first set in. The Pains came and went off at regular intervals, and were of sufficient force and frequency to induce the belief on the part of the attending Physician, that, though her Labor, from contracted Pelvis and the discharge of the Liquor Amnii so early, might be somewhat protracted, still that ultimate success would crown her efforts. Thus matters progressed for eighteen hours, when after an active Pain and a discharge of a quantity of Serous fluid, her Pains suddenly ceased, and with it all symptoms of Labor. I was immediately sent for and being eight miles distant and absent from home when the messenger arrived, I was delayed in seeing the patient until ten o'clock A. M. the next day, thirty hours after Parturition first set in. Upon an examination per Vagina, I found the soft parts not much dilated aud could feel no part of the child with my index finger, nor could I ascertain its true position after introducing my hand well lubricated as far up the Vagina as practicable. Upon an examination of the superficies of the abdomen, a prominence was detected which readily led to the conclusion that it was the child's head without the Uterus in the cavity of the Abdomen. This, in connection with the previous symptoms indicated to us clearly that the organ itself was ruptured, and that some active and decided treatment was necessary to the relief of the Patient. Such a case in twenty years' practice had not occurred to me. We immediately made known to the master the true condition of his servant and the probabilities of her recovery. We then called in Dr. Carey of Antioch, and upon consultation with Dr. Long and myself we determined that the operation of Gastrotomy was the only means by which she could possibly be relieved. Having determined upon the operation, the woman was placed upon a strong table upon her back, her feet drawn up to the nates, with a strong negro man to hold each hand and foot, I proceeded in the presence of Drs. Long and Carey to the operation. My first incision was along the Linea Alba, through the integument, from one inch below the Umbilicus to the Pubis. My second incision was through the abdominal muscles and Peritoneum into the cavity of the abdomen. The child's head presented at the upper part of the incision. I took hold of and delivered through the orifice a large well developed dead child. I tied and cut the Umbilical cord, and secured the end of it to a silver male catheter and passed it through the opening made by the incision into the Vagina, without the Vulva, loosed the fastening to the catheter, withdrew it, and delivered the Placenta per naturales vias. There was but little uterine hemorrhage. The woman manifested much fortitude; she neither moved hand or foot, nor did she complain at all during the whole course of the operation. Her pulse was full and round, never exceeding ninety to the minute. After the operation, I removed such of the clots from the abdomen as could not be removed with the hand, with castile soap-suds and a sponge, and closed the incision with seven sutures leaving an aperture in the Pubic region, through which any extraneous fluids might escape; between the sutures, adhesive slips were applied and over these, pledgets of Lint four or five double, wet with cold water were placed, and over all a broad bandage covering the whole abdominal parts, extending from the ensiform cartilage to the Pubis. We remained with the patient about three hours after the operation, and all were surprised to see her condition so favorable, after properly caring for her we left her with a promise to return the next day. We found her on the next day, about twenty four hours after the operation, doing well—the lochial discharge going on, her pulse about ninety, soft and round, her skin moderately relaxed, but her breathing somewhat hurried and a pain in her right side, we prescribed Oil and Turpentine applied a Sinapism over the seat of pain, which relieved her for the time. On the next day we found her with much pain, breathing hurried, no dejection from the bowels, her pulse very much accelerated, pain in her side had returned very acutely, which she described as a pleurisy pain—lochial discharge suspended. Ordered more Oil and Turpentine, with a stimulating enema every three hours until medicine operated. Removed the cold water dressing, and substituted warm water dressings as hot as she could bear them over the wound, and directed after her bowels should be thoroughly moved, that she should have four grs. of Dovers Power in a teacup half full of ginger tea, every three hours until the Lochia should be again established.

On our visit the next day we found her symptoms somewhat improved, her bowels had been actively moved, the Dovers Powder had brought about a return of the Lochia, her pulse not so frequent, but her breathing still hurried and the pain in her side still excruciating. She was cupped several times over the seat pf pain which relieved it greatly, and her breathing became almost natural. To check the hypercatharsis from the Oil, Laudanum, in doses of twenty-five drops every three hours until her bowels were controlled, was prescribed, and to keep up the secretion of milk, a puppy, which performed his part well, was applied to the breast, and the vagina washed out with the female syringe, twice per day with warm castile soap suds. On the ninth day after the operation, her condition was about as good as most females are at a like time in ordinary confinements, and she continued to improve from that time with but little medical treatment or trouble, save the dressing of the wound, until at the expiration of four weeks she had so far recovered as to be able to sit up and walk about.

She had a good getting up and no unfavorable symptoms with the exception of edema of the lower extremities, which readily yielded to the Roller Bandage and iron by hydrogen, and she is at this time entirely recovered and picks as much cotton daily as any other woman in the county.

*Remarks.*—In this perplexing case it became a point of much nicety to determine whether the patient should be left to the resources which nature might supply, or whether any means should be taken for relieving the abdomen from the presence of the fœtal body.

There are cases of reputed rupture of the Uterus on record in which the child has been left in the cavity of the abdomen and has been removed in a putrid state by abscess, the woman perfectly recovering. I am far from denying such a termination to these cases, but I should look upon them as most improbable, and I cordially coincide with Dewees in the opinion that almost all these cases have been instances of extra uterine conception and not of impregnation of the womb attended with rupture of the organ. Feeling as I did, that to leave the child in the cavity of the belly, was almost certain death to the mother, and she manifesting very great anxiety to have the operation performed, I determined to divide the parietes of the abdomen and to extract the child, rather than abandon the patient to the chance of what nature might effect.

I have thus given you a detailed account of the case referred to, with a hope that it may be interesting, if not edifying to the members of the Medical profession.

